# Identifying solutions to increase participation in physical activity interventions within a socio-economically disadvantaged community: a qualitative study

**DOI:** 10.1186/1479-5868-11-68

**Published:** 2014-05-23

**Authors:** Claire L Cleland, Ruth F Hunter, Mark A Tully, David Scott, Frank Kee, Michael Donnelly, Lindsay Prior, Margaret E Cupples

**Affiliations:** 1 UKCRC Centre of Excellence for Public Health (Northern Ireland), School of Medicine, Dentistry and Biomedical Sciences, Queen’s University Belfast, Clinical Sciences Block B, Royal Victoria Hospital, Grosvenor Road, Belfast BT12 6BJ, UK; 2 MRC/CSO Social and Public Health Sciences, University of Glasgow, Top floor, 200, Renfield Street, Glasgow G2 3QB, UK; 3 The School of Sociology, Social Policy and Social Work, Queen's University of Belfast, 6 College Park, Belfast, Northern Ireland BT7 1LP, UK; 4 Department of General Practice and Primary Care, Queen’s University Belfast, 1 Dunluce Avenue, Belfast BT9 7HR, UK

**Keywords:** Physical activity, Interventions, Socio-economic disadvantage, Community, Public health, Qualitative, Health information, Communication, Cross-sector working

## Abstract

**Background:**

There is an urgent need to increase population levels of physical activity, particularly amongst those who are socio-economically disadvantaged. Multiple factors influence physical activity behaviour but the generalisability of current evidence to such ‘hard-to-reach’ population subgroups is limited by difficulties in recruiting them into studies. Also, rigorous qualitative studies of lay perceptions and perceptions of community leaders about public health efforts to increase physical activity are sparse. We sought to explore, within a socio-economically disadvantaged community, residents’ and community leaders’ perceptions of physical activity (PA) interventions and issues regarding their implementation, in order to improve understanding of needs, expectations, and social/environmental factors relevant to future interventions.

**Methods:**

Within an ongoing regeneration project (Connswater Community Greenway), in a socio-economically disadvantaged community in Belfast, we collaborated with a Community Development Agency to purposively sample leaders from public- and voluntary-sector community groups and residents. Individual semi-structured interviews were conducted with 12 leaders. Residents (n = 113), of both genders and a range of ages (14 to 86 years) participated in focus groups (n = 14) in local facilities. Interviews and focus groups were recorded, transcribed verbatim and analysed using a thematic framework.

**Results:**

Three main themes were identified: *awareness of PA interventions; factors contributing to intervention effectiveness; and barriers to participation in PA interventions.* Participants reported awareness only of interventions in which they were involved directly, highlighting a need for better communications, both inter- and intra-sectoral, and with residents. Meaningful engagement of residents in planning/organisation, tailoring to local context, supporting volunteers, providing relevant resources and an ‘exit strategy’ were perceived as important factors related to intervention effectiveness. Negative attitudes such as apathy, disappointing experiences, information with no perceived personal relevance and limited access to facilities were barriers to people participating in interventions.

**Conclusions:**

These findings illustrate the complexity of influences on a community’s participation in PA interventions and support a social-ecological approach to promoting PA. They highlight the need for cross-sector working, effective information exchange, involving residents in bottom-up planning and providing adequate financial and social support. An in-depth understanding of a target population’s perspectives is of key importance in translating PA behaviour change theories into practice.

## Background

Increasing physical activity (PA) can improve health, reduce the risk of chronic disease and mortality and improve life expectancy [1-3]. Within higher income countries the importance of PA for health is of particular relevance among population groups with lower socio-economic status [4].

Research suggests that best efforts for PA promotion should focus on populations and the complex interactions among determinants of physical inactivity [5]. Multiple factors influence physical inactivity [6,7]. However, research into its determinants has focused mainly at an individual level [8]. Few studies have examined how its determinants may vary with socio-economic status or within socio-economically disadvantaged (SED) groups [9,10].

A recent review of PA interventions in SED communities identified that community-wide interventions had a small effect on increasing PA; group-based adult interventions were effective, individual interventions were not, but more evidence was required regarding the effectiveness of interventions targeting these ‘hard-to-reach’ communities [11]. Foster et al. [12] discussed the importance of identifying effective strategic approaches to increase recruitment of ‘hard-to-reach’ groups to PA interventions and the World Health Organisation [13] has identified a need to be proactive in overcoming difficulties in recruiting SED groups to studies. Further studies which include such groups would extend our knowledge and inform efficient and effective public health planning.

The social ecological model of behaviour change takes account of multiple levels of influences on people’s behaviour and provides a theoretical framework for developing an integrated approach to planning interventions to promote PA. It recognises the importance not only of an individual’s beliefs and capabilities but also of the physical environment within which they live and their community’s social and cultural norms [3]. Evidence suggests that key mediators of PA behaviour include self-efficacy and social support for PA [14]: these factors relate to the person and their environment and are significant elements of social ecological theory. Whilst many theoretical models of behaviour change have been constructed and (re)formulated by academics, comparatively little attention has been given within these to lay understandings of PA behaviour. Qualitative studies of lay perceptions about PA promotion are sparse, exposing the need to improve understanding about community relations and interactions, in order to ensure that theory is translated into practice and to design effective interventions [15].

There is also a need to develop successful approaches to tease apart complex interventions to identify their “active ingredients” and deal with component interactions [16,17]. The development of a complex intervention which can be generalised beyond the context of a specific study setting requires clear definition and detailed description of its various components. A mixed methods approach to its development, which includes qualitative data regarding its feasibility and relevance, is most likely to provide a design framework which can be applied to other settings.

Thus we aimed to explore, within a SED community, residents’ and leaders’ perceptions of PA interventions, intervention components and implementation strategies, in order to improve our understanding of needs, expectations, and factors relevant to future intervention design and delivery.

## Methods

The study was approved by the Office for Research Ethics Committees, Northern Ireland (09/NIR02/66).

### Study context and setting

The current study was carried out in the Connswater area of East Belfast and nested within the ‘Physical Activity and the Rejuvenation of Connswater’ (PARC) Study [18], which is a before-and-after evaluation of the effects of an ongoing urban regeneration project, the Connswater Community Greenway (CCG). When completed, the CCG will offer PA and active transport opportunities through environmental improvements (such as cycle and walkways). The study reported in this paper took place just prior to the implementation of environmental change and aimed to inform the development, design and delivery of PA interventions around the CCG.

The electoral wards comprising the study area are ranked within the lowest quintile of the Northern Ireland Multiple Deprivation Measure (NIMDM) [19], indicating that the area is socio-economically disadvantaged; 29.8% are economically inactive (Table 1). NIMDM scores are constructed by combining population data relating to seven different domains: income; employment; health deprivation and disability; education, skills and training; proximity to services; living environment; crime and disorder. NIMDM scores for wards range from 1 to 582. The study focus group participants lived in the following four wards: Ballymacarett, The Mount, Woodstock and Island; the NIMDM scores for these wards are 18, 25, 39 and 92 respectively.

**Table 1:** Demographic characteristics of focus group participants and background community

	**Focus group**		Community*	
	**Economic activity**			
	N (113)	%	N** (15,961)	%
Employed (full/part time or self- employed)	44	38.9	8250	52.6
Unemployed	22	19.5	1124	7.2
Retired	22	19.5	1638	10.4
Long term sick or disabled	11	9.7	1918	12.2
Full time student	12	10.6	1124	7.2
Looking after home or family	-	-	991	6.3
Other	-	-	646	4.1
Missing	2	1.8	-	-
	**Age group**			
	N (113)***	%	N* (20,958)	%
24 or younger	11	9.7	6889	32.8
25-34	17	15.0	4483	21.4
35-44	29	25.7	2987	14.3
45-54	23	20.4	2380	11.4
55-64	9	8.0	1665	7.9
65 plus	21	18.6	2554	12.2

*Ballymacarett, The Mount, Woodstock & Island Wards. Source: N Ireland Census 2011 http://www.nisra.gov.uk/Census/2011_results_local_characteristics.html; accessed 22/04/2014.

**Number aged 16-74 years who provided economic data.

***3 residents did not state their age.

The types of ‘interventions’ or programmes which had been delivered previously within the community ranged widely. They included ‘one-off’ walking and cycling events, walking groups which were mostly organised within existing community groups, indoor activities which targeted particular age groups (e.g. mothers of toddlers, or older people) and football, most notably for teenage males in a ‘midnight soccer’ league. Other interventions included leisure-centre based summer schemes for children and gym-based schemes for people with health-related conditions. Most publicity regarding these activities was delivered by posters, local news-sheets or word of mouth; information about major ‘one-off’ events was usually also communicated by radio or television advertising.

### Sample selection and recruitment

Community leaders who were invited to take part in interviews were leaders in statutory or voluntary organisations, who had experience of working in the wards in which the focus group participants lived and had a remit for planning or implementing PA interventions in the locality. They worked in charities, business organisations, community or social partnerships or in the local Council, Healthcare Trust, Education Board, Police Service or the Public Health Agency. The voluntary organisation leaders (4 male, 2 female) were aged 25 to 64 years; statutory organisation leaders (4 male, 2 female) were aged 35 to 65 years. Initially three leaders were selected purposively, to represent both sectors and different types of community groups. At the end of their interview they were each asked to identify another community leader who had experience of PA interventions, thus implementing a snowball sampling technique. All were invited via telephone or email.

Following completion and analysis of the interviews local community groups were contacted by an East Belfast Community Development Agency (EBCDA) representative or the CCG Community Engagement Officer to invite their participation in the study. Subsequently, with their agreement, a researcher (DS) contacted a group representative to provide further information and arrange a focus group (FG). This partnership approach facilitated recruitment and purposive sampling to reflect the diversity of gender, age and living situation within the study population. The FGs were conducted within local premises and at times designed to maximise convenience for residents. Those who invited residents to the groups were not asked to record information about people whom they invited but who declined to attend as this was considered to be a possible deterrent to recruitment and we did not seek ethical approval for this. Fourteen FGs were conducted, comprising of 113 participants aged 14-86 years (mean 45.3 years, SD 17.2); 57.5% (n = 65) were female, which was broadly reflective of the community’s gender distribution (51.5% female). Three groups included men only; one included teenage girls only; all others were of mixed age and gender. Overall, 55 (48.7%) were categorised as being economically inactive (unemployed, retired, disabled and “off sick”); 24 (21%) lived alone.

### Interview schedule

A flexible interview schedule was drafted based on previous literature and on the advice of CCG stakeholders regarding topics which were considered relevant to the local promotion of PA (Additional file 1). A semi-structured format ensured that relevant issues were covered whilst allowing researchers to probe interviewees. An iterative analysis process, with constant comparison of successive interviews, allowed questions to be refined in later interviews in order to explore evolving issues. Questions related to (1) perceptions/knowledge of current/past community PA interventions: (2) successful/unsuccessful intervention components; (3) perceived barriers/facilitators in promoting PA interventions.

A topic list, used in guiding FG discussions, was informed by a literature review [11] and consultation with PA experts and community leaders. It related to PA engagement; perceived PA barriers/facilitators and perceptions of PA interventions and the CCG.

### Data collection

Semi-structured one-to-one interviews were deemed the most appropriate method of gathering detailed information from community leaders regarding complex issues. All interviews were conducted in the interviewee’s place of work. Focus groups were chosen as the method by which the community residents’ views could best be explored, hoping that group dynamics would generate in-depth thinking and discussion. On arrival, participants completed a questionnaire to provide information about their demographic characteristics. Each interview (March-October 2010) and FG (December-January 2011) lasted approximately one hour. Two of three researchers were present at each interview (CC/RH/MT) and FG (DS/RH).

### Data management and analysis

With written informed consent, interviews and FGs were audio-recorded and transcribed anonymously. Transcripts of the interviews were analysed independently by two researchers; initial codes were identified and themes collated (CC/MC) [20,21]. In discussion with a third researcher (DS) these themes were reviewed and refined, ensuring clear definition. After 11 interviews, data saturation was achieved; another interview was conducted to seek confirmation of the analyses. FG data were analysed using an ‘a priori’ thematic framework, derived from the interview findings.

## Results

Three main themes were identified: 1) awareness of PA interventions; 2) factors contributing to intervention effectiveness; and 3) barriers to participation in PA interventions. Quotations to support the analysis were identified by a code, indicating their source: interviewee (IN), voluntary (V), statutory (S) or focus group (FG).

### Theme 1: Awareness of PA interventions

Interviewees highlighted a poor awareness of previous and current PA interventions, other than those in which they had personally been involved, suggesting a lack of interest among community leaders for the effective promotion of PA. Comments from both statutory and voluntary sector leaders reflected this lack of awareness.

“There are schemes [interventions] happening, but they’re not in east Belfast that I’m aware of and that’s the sad thing” (IN5S).

*“I don’t know ……..and that doesn’t mean to say that it’s not happening I just don’t know about them”* (*IN4V)*.

Their responses indicated that the nature of interventions varied between sectors. Voluntary sector interventions tended to focus on more general community activities and to include PA as a subsidiary component, thus potentially diluting the attention given to it. In contrast, statutory agencies’ relevant interventions focused on PA specifically and targeted specific groups.

“… holistic health and wellbeing programmes but would probably have an element of PA in them” (IN1V).

*“Healthwise Scheme….GP referral to the gym” [for people who are obese or have high blood pressure]”* (*IN5S).*

Comments reflected little linkage or shared communication between or within statutory or voluntary sectors or inclusion of local residents in the planning or delivery of initiatives. Leaders of voluntary organisations reported feeling *“out of the loop”* with statutory agencies, but also reported an absence of collaborative working between community groups.

“You’ve had this gap between the statutory and the non-statutory sector…there is not a huge amount of consultation with or community involvement. … They’ve all got their own programmes and they sort of guard them jealously, there’s very little in terms of joined up thinking........or strategic thinking” (IN2V).

These perceptions were shared by FG participants who were aware of poor co-ordination between organisers of various interventions. Their comments also indicated that residents failed to engage with interventions which they perceived as being personally irrelevant.

*“See if it doesn’t affect you and you don’t want to know,… leaflets in the houses advertising activities that are going on......, but people don’t want to know” (FG)*.

However, they were clear that if information, such as public health messages relating to PA, was perceived as being relevant to individuals within their community, then the strong socio-cultural influences which exist currently would ensure its effective dissemination.

“..... no matter what..... we find it out round here if we want to” (FG).

### Theme 2: Factors contributing to intervention effectiveness

#### ***Community engagement***

Interviewees perceived that there was value in involving members of the community from the outset of planning an intervention to ensure community “*ownership”* and to guarantee that plans would be relevant and tailored to the local context. Both voluntary and statutory sector leaders considered that it was important to target the *‘right people’* and to offer options regarding details of the intervention’s delivery, to help ensure that it would address people’s needs*.*

“…initiatives that have been successful have ....... taken into account where people are at and then have developed a programme accordingly” (IN1V).

FGs also emphasised the importance of community participation in developing acceptable plans and considering how to counter possible adverse influences for their implementation and the maintenance of facilities.

“Local organisations and groups are key.... need to involve....... give security to neighbourhood......respect privacy.... antisocial behaviour knowledge” (FG).

“If you get somebody involved in something they’re more likely to use it or they’re less inclined to break it” (FG).

#### ***Funding***

Both interviewees and FG participants acknowledged that securing funding is vital for the success of any intervention*.* The FGs highlighted the lack of readily available finance and identified that collaboration, rather than competition, would increase the likelihood of applications for funding being successful and gaining resources.

“Money, it’s as simple as that, you can build anything, you can make anything, you can run anything if you have the money” (IN5V).

“ … individual groups but not too many of them are working together and everybody’s trying to get their own funding and they’re all competing with each other” (FG).

However, it was recognised that money alone would not ensure success: several interviewees emphasised how community engagement and volunteer support was necessary for interventions to attract participants. They also emphasised that community volunteers needed to be “*looked after”,* to foster their continuing involvement.

“Making use of volunteers and their time; it’s key to any of these initiatives” (IN1V).

#### ***Strategic planning***

Interviewees recognised a need for strategic planning, and better linkage between organisations and with the community. They perceived that it was important to have an identified *“exit strategy”*, whereby interventions promoting PA were not isolated short-term events but were planned to support a sustained change of behaviour, with resources that would be available in the longer term.

“An exit route….extremely important too, in that we try as best possible to put in place and to target activities where that input can be sustained” (INV5).

It was suggested that a multi-sectoral interdisciplinary team, which worked collaboratively and shared information would allow more informed planning in an ongoing context with “*very much reduced duplication”* and more effective use of resources.

### Theme 3: Barriers to participation in PA interventions

#### ***Apathy***

Apathy was identified as a barrier to PA promotion. When communities were informed about proposed interventions which were not implemented there was a loss of interest in subsequent proposals.

*“Bombarded with potential programmes….overworked or jaded or maybe a wee bit burnt out”* (*IN1V).*

“Over-saturation problem …” (IN9S).

Apathy was also linked to poor self-esteem among individuals within the community and leaders suggested that there was a need for specific programmes to support the development of personal skills. Residents felt they needed *“encouragement”* to become involved in community interventions; their comments reflected disappointment, to which they appeared resigned rather than surprised, at the failure of community leaders to follow-up initial discussions regarding a succession of suggested initiatives.

“… *a lot of low self-esteem, personal development is much needed” (IN4V).*

“…you hear one story this week and then a few weeks later another story appears in the press and then another one .........” (FG).

#### ***Lack of facilities***

Interviewees from voluntary organisations reported that it was difficult to access resources for interventions and considered that having established facilities within the locality would promote engagement in PA in the longer term.

“.. like if you want something that is going to be sustained and continued on, having a resource base in place obviously helps” (IN9S).

FGs also identified difficulties relating to practical facilities (toilets and seats), lack of services (child-minding and carer support) and fears for safety (due to poor lighting and inadequate supervision/security), emphasising the importance of involving local residents to identify specific needs and appropriate plans to address these in specific localities.

#### ***Information dissemination***

Interviewees felt the community lacked knowledge of the benefits of PA for health. They also felt there was a need to have, based in the locality, someone who was trained to support people who wished to become more active. They appeared to recognise a communication gap between themselves and the community in their current approaches to promoting PA and to perceive inadequacy in their own knowledge. However, they showed no recognition of individuals’ personal difficulties in becoming physically active. No comments reflected any awareness of personal, socio-cultural or physical barriers to PA or a sense of understanding of the community’s perceptions of the value of PA.

“There’s some sort of step we need to take to get into people’s mind the importance of PA and how easy PA can be” (IN4V).

“….some kind of local campaign linking in with like a regional based campaign identifying the benefits of PA” (IN8S).

“You’re going to start off telling people first, then you’re going to remind them, then you’re going to re-remind them” (IN5S).

FGs highlighted the importance of face-to-face contacts and social-networking in communication. Personal contacts, involving *‘word of mouth’,* were perceived to be the most frequent and effective method of disseminating information. Residents also highlighted how they valued being kept informed about the ongoing progress of interventions and that this encouraged community engagement.

“Keep people up-to-date with how it’s going” (FG).

## Discussion

This study highlights the existence of poor intra- and inter-sector communication, with ineffective sharing of information regarding PA interventions in SED communities. It also shows a need for better recognition, by service providers, of problems in people’s physical and socio-cultural environments which hinder their active engagement and participation in PA interventions.

### Communication and partnership

Both leaders and residents revealed poor awareness of current and past interventions and poor communication and collaboration between and within organisations. The WHO highlighted the importance of inter-sectoral working and sharing knowledge, to reduce health inequalities [22]. Our findings suggest further work should be performed to establish mechanisms for inter-sectoral collaboration in policy making [23] and for promoting partnerships [13] in policies and planning. Evaluation frameworks which assess inter-sectoral working encourage its effective implementation at the level of countries [24]. The use of formal evaluations of communication networks may be effective in increasing the promotion and uptake of PA initiatives at a community level.

In previous work Burgoyne et al. [25] reported low levels of community awareness of PA interventions; residents in that study admitted that information was available but considered that it was poorly disseminated. Our leaders’ admissions of poor awareness of PA interventions may reflect a failure of service providers to prioritise or recognise the importance of PA for health. Residents expressed strong views that if they wanted to find out more information about PA interventions they could and would do so. Thus, our findings emphasise the need to understand the socio-cultural context of target groups and to tailor information to capture their interest. When planning interventions, consideration should be given to the multiple factors which influence PA perceptions and behaviour: the use of a systematic theoretical framework, such as the behaviour change wheel [26], is recommended.

### Community engagement

Our findings provide examples of the value of involving residents and they support recent recommendations to engage target communities from the outset in PA initiatives [13,23]. They also show how public engagement which identifies local problems and illuminates community perceptions could inform research development [27] and the design of interventions which target disadvantaged groups [13].

Unsatisfactory experiences of engagement in previous initiatives had a negative impact on residents’ readiness to engage in future interventions; actively involving them in planning and delivery should facilitate communications with service providers and support their long-term engagement in projects. A recent review [28] suggests that building meaningful partnerships of diverse communities can improve health outcomes. Social support and self-efficacy have been identified as key mediators of PA participation in disadvantaged areas [14] and our qualitative findings illustrate their relevance.

### Needs and resources to support PA interventions

Multiple factors contribute to individuals’ capability, opportunity, and motivation to change their PA [26]. Giving communities a sense of ownership should empower individuals and increase their capabilities. Multi-level interventions [14] and multidisciplinary teamwork are needed, in order to allow strategic planning and avoid unnecessary duplication of effort and resources. There is also a need to revise the content and delivery of PA public health messages, to assure disadvantaged communities of their personal relevance for them. PA information delivered to SED groups should be ‘clear and consistent’, enhancing confidence and beliefs [6]. Sharing community leaders’ and lay residents’ knowledge with those who plan PA interventions should optimise opportunities, ensure safety and provide appropriate facilities for all. Leaders identified a need for a skilled community resource, to provide residents with PA advice. Increasing residents’ awareness of the relevance of PA for health may promote collaboration between community groups, and success in their applications for funding [29].

### Strengths and limitations

This study provides a rich resource of perceptions of PA initiatives in a SED community. Its focus is on informing the development of successful interventions to address a major public health problem for a population sub-group, rather than on exploring personal barriers and facilitators to the uptake of PA opportunities. Researchers determined independently that data saturation was achieved and findings were corroborated in FGs. This study provides recruitment details and participant demographics, which set our findings in context and inform their interpretation. All leaders who were invited and all groups which were invited agreed to collaborate.

We recognise that our findings may not represent the views of non-participants and that those who took part in the interviews and FGs may have been leaders and residents with a particular interest in PA. We used a snowball sampling technique to recruit leaders, so that it is possible that only those with a similar interest in PA were identified for invitation. However, we were careful to gather information regarding both successful and unsuccessful interventions, in order to ensure that we heard about as wide a variety of experiences as possible. We did not have ethical permission to gather background data regarding residents who were invited and did not participate but our FG data included participants’ reports of views expressed by others in the wider community and of how they had engaged in previous interventions. In comparison with the background community (Table 1), FGs included similar proportions of males and females, people whose ages ranged from teenage to over 80 years, and people who were employed, unemployed, retired and ‘long-term sick’ but did not include those who reported significant caring responsibilities. We acknowledge that our evidence may not be directly transferable to other settings or groups.

## Conclusions

This study supports a social-ecological approach to the development and delivery of PA interventions and highlights how PA promotion is complex, influenced by a multitude of factors. It adds to existing knowledge regarding sources of complexity within multi-component PA interventions and adds depth to our understanding of how different components may interact [16,17]. It identifies “active” intervention “ingredients” which may be applied to future PA intervention design and development [16,17] (Additional file 2).

In relation to the specific context of the current study, the researchers have actively engaged with local community partnerships, city council, health practitioners and government departments and have planned an evaluation framework for the regeneration project [18]. Leaders have recognised their need to forge links with colleagues, other groups and residents and to plan coordinated approaches to promote behaviour change, following theory-based intervention mapping [30].

SED populations may have difficulties in developing self-efficacy and acquiring attitudes, skills and access to facilities that enable PA participation. With community involvement from the outset, interventions and public health messages can be tailored to ensure their understanding, relevance and accessibility for people living within that community. However, more work is needed to inform the best approach for effective knowledge transfer regarding the importance of PA for health and well-being and to effect behaviour change in increasing PA in SED communities.

## Abbreviations

CC: Claire Cleland; CCG: Connswater Community Greenway; DS: David Scott; EBCDA: East Belfast Community Development Agency; FG: Focus Group; FK: Frank Kee; HSC: Health and Social Care; IN: Interviewee; LP: Lindsay Prior; MC: Margaret Cupples; MD: Michael Donnelly; MDM: Multiple Deprivation Measure; MT: Mark Tully; NFA: No Fixed Abode; NICE: National Institute for Health and Care Excellence; NIMDM: Northern Ireland Multiple Deprivation Measure; NISRA: Northern Ireland Statistical Research Agency; PA: Physical Activity; PARC: Physical Activity and the Rejuvenation of Connswater; R&D: Research & Development; RH: Ruth Hunter; S: Statutory; SED: Socio-economically disadvantaged; UKCRC: United Kingdom Clinical Research Collaboration; V: Voluntary; WHO: World Health Organisation; Yrs: Years.

## Competing interests

The authors declare that there are no conflicts of or competing interests.

## Authors’ contributions

CC made a substantial contribution to the design of the study, conducted the interviews, analysed the data and drafted the manuscript. RH contributed to the design, data collection and analysis. DS made a substantial contribution to the design of the focus group arm of the study, conducted the focus groups and analysed the data. FK, MT, MC, LP and MD conceived and contributed to the design of the study and data analysis. MT also contributed to the data collection. MC also led the revision process for the manuscript. All authors have contributed to the revision of successive drafts of the paper and approved the final manuscript and have agreed to be accountable for all aspects of the work in ensuring that questions related to the accuracy or integrity of any part of the work are appropriately investigated and resolved.

## Supplementary Material

Additional file 1 Interview schedule. Click here for file

Additional file 2 Checklist for the design and development of physical activity interventions in socio-economically disadvantaged communities. Click here for file
