# Supplementary material for: Identifying solutions to increase participation in physical activity interventions within a socio-economically disadvantaged community: a qualitative study
Source: Int J Behav Nutr Phys Act. 2014 May 23;11:68. doi: 10.1186/1479-5868-11-68 (PMC4038056; doi:10.1186/1479-5868-11-68)
Supplement: Additional file 1 — Interview schedule. [file 1479-5868-11-68-S1.docx]

**Appendix 1 – Interview schedule**

Introduction

- Who we are; QUB and in terms of the PARC study.
- Conducting interviews with key individuals involved in East Belfast to identify target groups, barriers and facilitators to promote physical activity (PA) in Connswater.
- The data collected will be audio recorded (interviewee will be kept anonymous) and analysed with the intention of using it to inform future focus group discussion and written up as part of a PhD thesis.

**Interview Questions**

**Benefits and importance of physical activity**

1. What do you feel are the benefits of becoming physical active or undertaking more PA?

- Impact on health: individual, group, community (different ages)
- Mental health: individual, group, community
- Socialising
- Forming habits

1. How important do you feel PA promotion is within the remit of your organisation?
2. How important is PA within the other organisations and community groups in East Belfast, on an organisational level?

**Current PA promotion initiatives/schemes**

1. Do you know of any other PA initiatives/schemes that are running in the East Belfast area?

- Who is taking the lead on setting up these initiatives/schemes?
- What groups are running these initiatives?
- How long have they been running?
- What numbers of participants are involved?
- What resources are they using?
- Who is resourcing it?

(*If a large number of initiatives/schemes are identified, then ask interviewee to discuss in more detail 5-10 key ones)

2. In your opinion what features make initiatives/schemes successful?

- Why?
- What makes them effective?
- What features do you feel make (or would make) these programmes sustainable in the long term?
- Barriers?

3. In your opinion what features have made schemes unsuccessful?

- Why?
- What features do you feel made (or would make) these programmes unsuccessful in being sustained long term?

4. Learning from these previous initiatives/schemes, what is required in the future to make PA promotion initiatives/schemes sustainable for the long term?

**Facilitators**

1. What do you think would work in terms of encouraging PA and PA promotion initiatives in East Belfast?

- What resources are needed (£/equipment/activities/materials)
- Types of activities (e.g. in the workplace/school)
- Mode of delivery (how/who)
- Promotion (community based social marketing; internet; local radio; mobile phone; or local agencies e.g. church)
- Government policies and Belfast City Council
