# Supplementary material for: Identifying solutions to increase participation in physical activity interventions within a socio-economically disadvantaged community: a qualitative study
Source: Int J Behav Nutr Phys Act. 2014 May 23;11:68. doi: 10.1186/1479-5868-11-68 (PMC4038056; doi:10.1186/1479-5868-11-68)
Supplement: Additional file 2 — Checklist for the design and development of physical activity interventions in socio-economically disadvantaged communities. [file 1479-5868-11-68-S2.docx]

**Appendix 2 - Checklist for the design and development of physical activity interventions in socio-economically disadvantaged communities**

| **Number** | **Guidance/components** | | **Included (tick)** |
| --- | --- | --- | --- |
| 1 | Inter-sectoral steering group for strategic planning | Statutory sector representation |  |
|  |  | Voluntary sector representation |  |
|  |  | Community residents |  |
| 2 | Identify theoretical framework for intervention development | |  |
| 3 | Establish knowledge sharing pathway within/ between organisations | |  |
| 4 | Involve community | Concept development |  |
|  |  | Design (address specific needs) |  |
|  |  | Intervention development |  |
|  |  | Secure funding |  |
|  |  | Share information |  |
|  |  | Recruitment |  |
|  |  | Delivery/ implementation |  |
| 5 | Engage volunteer support: ensure intervention information, design and resources are relevant to individuals in community | |  |
| 6 | Train community volunteers/champions to provide relevant advice on health and physical activity | |  |
| 7 | Establish an exit strategy | |  |
| 8 | Foster ongoing community support: ensure feedback/ involvement in further planning/ support development of personal skills | |  |
